# Biofilm and planktonic pneumococci demonstrate disparate immunoreactivity to human convalescent sera

**DOI:** 10.1186/1471-2180-11-245

**Published:** 2011-11-02

**Authors:** Carlos J Sanchez, Brady J Hurtgen, Anel Lizcano, Pooja Shivshankar, Garry T Cole, Carlos J Orihuela

**Affiliations:** 1 Department of Microbiology and Immunology, The University of Texas Health Science Center at San Antonio, San Antonio, TX 78229, USA; 2 Department of Biology and South Texas Center for Emerging Infectious Diseases, University of Texas at San Antonio, San Antonio, TX 78249, USA

## Abstract

**Background:**

*Streptococcus pneumoniae *(the pneumococcus) is the leading cause of otitis media, community-acquired pneumonia (CAP), sepsis, and meningitis. It is now evident that *S. pneumoniae *forms biofilms during nasopharyngeal colonization; the former which facilitates persistence, the latter, a prerequisite for subsequent development of invasive disease. Proteomic evaluation of *S. pneumoniae *suggests the antigen profile available for host-recognition is altered as a consequence of biofilm growth. This has potentially meaningful implications in regards to adaptive immunity and protection from disseminated disease. We therefore examined the antigen profile of biofilm and planktonic pneumococcal cell lysates, tested their reactivity with human convalescent sera and that generated against biofilm pneumococci, and examined whether immunization with biofilm pneumococci protected mice against infectious challenge.

**Results:**

Biofilm pneumococci have dramatically altered protein profiles versus their planktonic counterparts. During invasive disease the humoral immune response is skewed towards the planktonic protein profile. Immunization with biofilm bacteria does not elicit a strong-cross-reactive humoral response against planktonic bacteria nor confer resistance against challenge with a virulent isolate from another serotype. We identified numerous proteins, including Pneumococcal serine-rich repeat protein (PsrP), which may serve as a protective antigens against both colonization and invasive disease.

**Conclusion:**

Differential protein production by planktonic and biofilm pneumococci provides a potential explanation for why individuals remain susceptible to invasive disease despite previous colonization events. These findings also strongly suggest that differential protein production during colonization and disease be considered during the selection of antigens for any future protein vaccine.

## Background

*Streptococcus pneumoniae *(the pneumococcus) is the leading cause of otitis media, community-acquired pneumonia (CAP), sepsis, and meningitis. Primarily a commensal, *S. pneumoniae *colonizes the nasopharynx of 20-40% of healthy children and 10-20% of healthy adults. In most instances nasopharyngeal colonization is asymptomatic and self-limited. However, in susceptible individuals, in particular infants and the elderly, *S. pneumoniae *is capable of disseminating to sterile sites and causing opportunistic invasive disease [1-4]. Worldwide and despite aggressive vaccination policies, the pneumococcus is responsible for approximately 1.6 million childhood deaths per year and is associated with a case-fatality rate exceeding 20% in individuals >65 years of age [5-7]. Thus, the disease burden caused by the pneumococcus is tremendous.

It is now evident that *S. pneumoniae *forms biofilms during colonization and in the middle ear during otitis media. Pneumococcal biofilms have been detected in the nasopharynx and sinuses of individuals with chronic rhinosinusitis, the surface of resected adenoids, occluded tympanostomy tubes and mucosal epithelial cells isolated from the middle-ear of children with persistent otitis media, and biofilm aggregates have been observed in nasal lavage fluids collected from experimentally infected mice [8-14]. In general, bacterial biofilms are a community of surface-attached microorganisms that are surrounded by an extracellular polymeric matrix (EPM) composed of DNA, polysaccharide, and protein [15-17]. Due to their EPM, as well as altered gene transcription, metabolism, and growth rate, biofilm pneumococci have been shown to be resistant to desiccation, host mechanisms of clearance including opsonophagocytosis, and to antimicrobial therapy [14,16,18-22]. Thus, growth within a biofilm presumably facilitates *S. pneumoniae *persistence during colonization. A notion supported by the finding that *S. pneumoniae *mutants deficient in biofilm formation *in vitro *were outcompeted by wild type bacteria in the nasopharynx of mice [23].

Proteomic evaluation of a serotype 3 *S. pneumoniae *clinical isolate found that the protein profile between planktonic exponential growth-phase bacteria and those in a mature biofilm differed by as much as 30% [24]. Numerous investigators have since shown biofilm-dependent changes in gene-expression and the production of established virulence determinants. These include the candidate protein vaccine antigens: pneumolysin, a cholesterol-dependent cytolysin [25]; pneumococcal serine-rich repeat protein (PsrP), a lung cell and intra-species adhesin [14,26,27]; choline binding protein A (CbpA), an adhesin required for colonization and translocation across the blood brain barrier [28,29], and pneumococcal surface protein A (PspA), an inhibitor of complement deposition [23,30,31]. Thus, the antigen profile available for host-recognition is altered as a consequence of the mode of bacterial growth (i.e. biofilm versus planktonic growth) with potentially meaningful implications in regards to adaptive immunity.

For the latter reason, we examined the antigen profile of biofilm and planktonic pneumococcal cell lysates and tested their reactivity with human convalescent sera. Additionally, we examined whether antibodies generated against biofilm pneumococci preferentially recognized cell lysates from either the planktonic or biofilm phenotype and protected against infectious challenge. Our findings show that the humoral immune response developed during invasive disease is strongly skewed towards the planktonic phenotype. Furthermore, that the antibody response generated against biofilm bacteria poorly recognizes planktonic cell lysates and does not confer protection against virulent pneumococci belonging to another serotype. These findings provide a potential explanation for why individuals remain susceptible to invasive disease despite prior colonization and strongly suggest that differential protein production during colonization and disease be considered during the selection of antigens for any future vaccine.

## Results

### Differential protein production during biofilm growth

**Figure 1:**
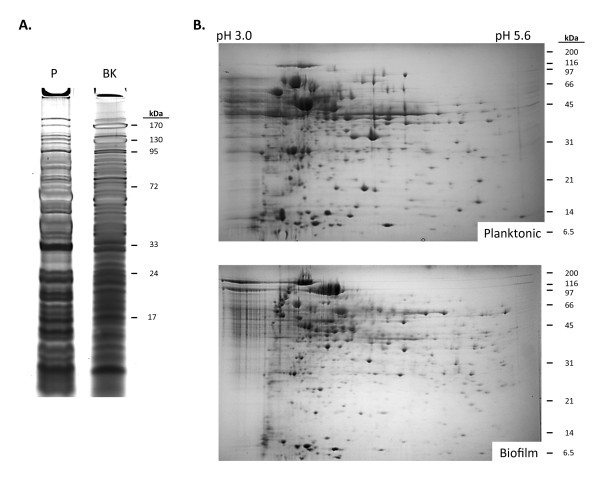
**Comparison of protein expression profiles of planktonic and mature *S. pneumoniae *biofilms**. **A) **Crude protein extracts (50 μg) of *S. pneumoniae *grown under planktonic (PK) or biofilm (BF) conditions separated by SDS-PAGE using 12% polyacrylamide gels and silver stained. **B) **Representative 2DGE images of total cell lysates of *S. pneumoniae *TIGR4 grown planktonically and as a 2 day old biofilm. Crude protein extracts (300 μg) were separated on pH 3.0-5.6 Immobiline Dry strips followed by SDS-PAGE using 8-16% polyacrylamide gels. Gels were stained with Coomassie blue.

Large-scale proteomic analysis of *S. pneumoniae *during biofilm growth is currently limited to a single isolate, serotype 3 strain A66.1 [24]. To examine the protein changes incurred during mature biofilm growth in TIGR4, a serotype 4 isolate, we first separated cell lysates from planktonic and biofilm TIGR4 by 1DGE and visualized proteins by silver stain (Figure 1A). As would be expected, extensive differences were observed with numerous unique protein bands present in either the biofilm or planktonic lanes, some bands with enhanced intensity under one growth condition, and other bands demonstrating no change. Following visualization of whole cell lysates by 2DGE and Coomassie blue staining, we confirmed biofilm-growth mediated changes at the individual protein level with numerous spots having reproducible unique and enhanced/diminished protein spots the gels (Figure 1B).

To identity those proteins with altered biofilm production, whole cell lysates from biofilm and planktonic pneumococcal cell lysates were separated by 1DGE and proteins within the gel were identified by MALDI-TOF analysis by cross-referencing the detected peptides against the TIGR4 genome. Of note, enumeration of the detected peptides allows for a semi-quantitative analysis [32], thus we could assess whether the detected proteins were altered during biofilm growth. In total, 123 proteins met our stringent criteria for identification (see methods), 103 (84%) of which demonstrated a ≥2-fold difference in the number of enumerated peptides in a given growth-phenotype (Table 1). Strikingly, during biofilm versus planktonic growth, 96 proteins (78%) had diminished production and only 8 proteins (6.5%) had enhanced production. The former included proteins involved in mRNA translation (i.e. Elongation factor Tu and G, 50 s and 30 s ribosomal proteins), virulence (i.e. pneumolysin, enolase, pyruvate oxidase) and assorted metabolic pathways. Thus our findings were in agreement with the overall accepted notion that biofilm bacteria experience reduced protein synthesis, altered virulence determinant production, and have an altered metabolism [15,16]. The 8 proteins found to be upregulated during TIGR4 biofilm growth included: PsrP; Foldase protein A (PrsA); the manganese ABC transporter PsaA; ArcB, an ornithine carbamolytransferase; AsnA, an asparate ammonia ligase subunit; the CTP synthase PyrG; PrfC, a peptide chain release factor; and SP_0095, a protein with unknown function.

**Table 1:** Comparison of Protein Expression Profiles during Biofilm and Planktonic Growth

			Detected Peptides	
			
Group and Function	Protein	Gene	Biofilm	Planktonic
Protein synthesis and processing	30S ribosomal protein S2	*rpsB *(SP_2215)	14	22
	30S ribosomal protein S3	*rpsC *(SP_0215)	4	5
	30S ribosomal protein S4	*rpsD *(SP_0085)	4	5
	30S ribosomal protein S5	*rpsE *(SP_0227)	6	23
	30S ribosomal protein S8	*rpsH *(SP_0224)	6	10
	30S ribosomal protein S7	*rpsG *(SP_0272)	0	3
	30S ribosomal protein S10	*rpsJ *(SP_0208)	3	4
	30S ribosomal protein S12	*rpsL *(SP_0271)	0	3
	30S ribosomal protein S11	*rpsK *(SP_0235)	4	6
	30S ribosomal protein S13	*rpsM *(SP_0234)	0	2
	30S ribosomal protein S17	*rpsQ *(SP_0218)	0	4
	50S ribosomal protein L1	*rplA *(SP_0631)	9	28
	50S ribosomal protein L2	*rplB *(SP_0212)	0	7
	50S ribosomal protein L3	*rplC *(SP_0209)	0	4
	50S ribosomal protein L4	*rplD *(SP_0210)	5	11
	50S ribosomal protein L5	*rplE *(SP_0221)	10	23
	50S ribosomal protein L6	*rplF *SP_0225	6	5
	50S ribosomal protein L7/L12	*rplL *(SP_1354)	0	14
	50S ribosomal protein L9	*rplI *(SP_2204)	0	2
	50S ribosomal protein L10	*rplJ *(SP_1355)	0	7
	50S ribosomal protein L11	*rplK *(SP_0630)	0	9
	50S ribosomal protein L14	*rplN *(SP_0219)	3	5
	50S ribosomal protein L15	*rplO *SP_0229	0	5
	50S ribosomal protein L18	*rplR *(SP_0226)	0	3
	50S ribosomal protein L19	*rplS *(SP_1293)	8	8
	50S ribosomal protein L21	*rplU *(SP_1105)	0	2
	50S ribosomal protein L22	*rplV *(SP_0214)	0	6
	50S ribosomal protein L30	*rpmD *(SP_0228)	0	2
	Elongation factor G	*fusA *(SP_0273)	10	72
	Elongation factor P	*efp *(SP_0435)	5	7
	Elongation factor Ts	*tsf *(SP_2214)	14	16
	Elongation factor Tu	(SP_0681)	29	59
	Arginyl-tRNA synthetase	*argS *(SP_2078)	11	14
	Alanyl-tRNA synthetase	*alaS *(SP_1383)	4	8
	Glutamyl-tRNA(Gln) amidotransferase subunit A	*gatA *(SP_0437)	0	4
	Glycyl-tRNA synthetase alpha subunit	*glyQ *(SP_1475)	0	4
	Methionyl-tRNA formyltransferase	*fmt *(SP_1735)	0	2
	Methionyl-tRNA synthetase	*metG *(SP_0788)	0	2
	Phenylalanyl-tRNA synthetase beta chain	*pheT *(SP_0581)	0	2
	Prolyl-tRNA synthetase	*proS *(SP_0264)	0	12
	10 kDa chaperonin	*groES *(SP_1907)	3	8
	60 kDa chaperonin	*groEL *(SP_1906)	24	57
	33 kDa chaperonin	*hslO *(SP_2188)	0	4
	Chaperone protein dnaK	*dnaK *(SP_0517)	12	89
	ATP-dependent Clp protease ATP-binding subunit	*clpE *(SP_0820)	3	3
	ATP-dependent Clp protease proteolytic subunit	*clpP *(SP_0746)	0	5
	Adapter protein mecA	mecA SP_1362	0	2
	Ribosome-recycling factor	*frr *(SP_0945)	0	2
	Foldase protein prsA	*prsA *SP_0981	13	5
	Peptide chain release factor 3	*prfC *(SP_0439)	4	0
	Peptide deformylase	*def *(SP_1456)	0	4
	Protein grpE	*grpE *(SP_0516)	0	5
				
**Energy Metabolism**	Phosphoglycerate kinase	*pgk *(SP_0499)	22	46
	L-lactate dehydrogenase	*ldh *(SP_1220)	21	30
	Glyceraldehyde-3-phosphate dehydrogenase	*gapN *(SP_1119)	10	38
	Fructose-bisphosphate aldolase	*fba *SP_0605	6	32
	Glycerol-3-phosphate dehydrogenase [NAD(P)+]	*gpsA *(SP_2091)	2	6
	2,3-bisphosphoglycerate-dependent phosphoglycerate mutase	*gpmA *(SP_1655)	7	11
	6-phosphofructokinase	*pfkA *(SP_0896)	8	21
	Phosphoenolpyruvate-protein phosphotransferase	*ptsI *(SP_1176)	0	10
	Ribose-phosphate pyrophosphokinase 1	*prsA *(SP_0027)	2	4
	Ribose-5-phosphate isomerase A	*rpiA *(SP_0828)	0	3
	Triosephosphate isomerase	*tpiA *(SP_1574)	2	8
	Tagatose 1,6-diphosphate aldolase	*lacD *(SP_1190)	4	6
	Phosphoenolpyruvate-protein phosphotransferase	*ptsI (*SP_1176)	0	6
	Ribose-phosphate pyrophosphokinase 2	*prs2 *(SP_1095)	0	3
	Phosphoglucosamine mutase	*glmM *(SP_1559)	0	2
	Glucosamine--fructose-6-phosphate aminotransferase [isomerizing]	*glmS *(SP_0266)	12	31
	Ornithine carbamoyltransferase, catabolic	*arcB *(SP_2150)	4	2
	Dihydrodipicolinate reductase	*dapB *(SP_1555)	0	4
	Dihydrodipicolinate synthase	*dapA *(SP_1014)	0	3
	Glucosamine-6-phosphate deaminase	*nagB *(SP_1415)	0	2
	Carbamoyl-phosphate synthase large chain	*carB *(SP_1275)	5	5
	2,3,4,5-tetrahydropyridine-2,6-dicarboxylate N-acetyltransferase	*dapH *(SP_2097)	0	8
	Aspartate--ammonia ligase	*asnA *(SP_1970)	3	0
	Dihydroxy-acid dehydratase	*ilvD *(SP_2126)	0	7
	ATP synthase subunit alpha	*atpA *(SP_1510)	4	21
	ATP synthase subunit beta	*atpD *(SP_1508)	9	18
	ATP synthase gamma chain	*atpG *(SP_1509)	0	8
	Phosphate import ATP-binding protein PstB 1	*pstB1 *(SP_1396)	0	9
	Phosphate import ATP-binding protein PstB 2	*pstB2 *(SP_1397)	0	4
	Maltose/maltodextrin-binding protein	*malX *(SP_2108)	0	10
	Manganese ABC transporter substrate-binding lipoprotein	*psaA *(SP_1650)	13	0
	GMP synthase [glutamine-hydrolyzing]	*guaA *(SP_1445)	6	21
	Hypoxanthine-guanine phosphoribosyltransferase	*guaA *(SP_1445)	0	9
	Adenylate kinase	*guaA *(SP_1445)	2	5
	Inosine-5'-monophosphate dehydrogenase	*guaA *(SP_1445)	0	14
	Uracil phosphoribosyltransferase	*guaA *(SP_1445)	4	11
	Dihydroorotate dehydrogenase	*guaA *(SP_1445)	0	5
	Uridylate kinase	*guaA *(SP_1445)	0	5
	CTP synthase	*guaA *(SP_1445)	3	0
	Bifunctional protein glmU	*guaA *(SP_1445)	0	3
	Acetyl-coenzyme A carboxylase carboxyl transferase subunit beta	*accD *(SP_0426)	0	2
	Phosphate acyltransferase	*plsY *(SP_0851)	0	2
	Formate--tetrahydrofolate ligase	*fhs *(SP_1229)	0	5
	6,7-dimethyl-8-ribityllumazine synthase	*ribH *(SP_0175)	0	3
	manganese-dependent inorganic pyrophosphatase	*ppaC *(SP_1534)	2	3
	Serine hydroxymethyltransferase	*glyA *(SP_1024)	0	7
	Pyridoxal biosynthesis lyase pdxS	*pdxS *(SP_1468)	0	9
				
**Capsule production & Cell wall**	Tyrosine-protein kinase CpsD	*cpsD *(SP_0349)	0	2
	Glucan 1,6-alpha-glucosidase	*dexB *(SP_0342)	2	3
	UTP--glucose-1-phosphate uridylyltransferase	*cap4C *(SP_2092)	4	4
	UDP-N-acetylmuramoylalanine--D-glutamate ligase	*murD *(SP_0688)	0	3
	D-alanine--poly(phosphoribitol) ligase subunit 1	*dltC *(SP_2174)	0	3
				
**Virulence Factors**	Enolase	*eno *(SP_1128)	25	89
	Pyruvate oxidase	*spxB *(SP_0730)	28	62
	Pneumolysin	*ply *(SP_1923)	0	11
	PsrP	*psrP *(SP_1772)	72	21
				
**Unknown & Hypothetical**	DegV domain-containing protein	(SP_1112)	0	5
	UPF0176 protein	(SP_0095)	2	0
	UPF0371 protein	(SP_0341)	0	6
	UPF0082 protein	(SP_1922)	0	8
	Probable transketolase	*tkt *(SP_2030)	4	37
				
**Regulation & DNA Binding**	HPr kinase/phosphorylase	*hprK *(SP_1413)	0	2
	Single-stranded DNA-binding protein	*ssb *(SP_1540)	0	9
	DNA-binding protein HU	*hup *(SP_1113)	0	10
	GTP-sensing transcriptional pleiotropic repressor CodY	*codY *(SP_1584)	0	2
	Pur operon repressor	*purR *(SP_1979)	0	5
				
**Transcription**	DNA-directed RNA polymerase subunit alpha	*rpoA *(SP_0236)	5	3
	DNA-directed RNA polymerase subunit beta	*rpoB *(SP_1961)	2	4
	Transcription elongation factor GreA	*greA *(SP_1517)	0	3

### Biofilm and planktonic pneumococci have disparate immunoreactivity with antiserum

**Figure 2:**
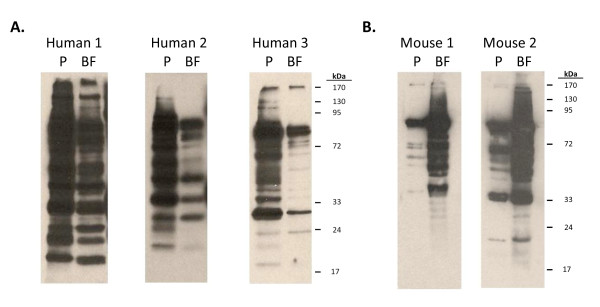
**Human convalescent sera has diminished reactivity against proteins from biofilm pneumococci**. Whole cell lysates from biofilm (BF) and planktonic (PK) pneumococci were separated by 1DGE and transferred to nitrocellulose. Membranes were probed using **A) **convalescent sera from humans recovered from confirmed pneumococcal pneumonia or **B) **sera from mice immunized with biofilm pneumococci.

To determine whether these growth-phase dependent changes altered the immunoreactivity of pneumococci, we compared the ability planktonic and biofilm TIGR4 cell lysates to react with convalescent sera from humans who had confirmed pneumococcal pneumonia and sera from mice immunized with ethanol-killed *S. pneumoniae *biofilm pneumococci. Following immunoblotting with human convalescent sera, robust detection of proteins in the planktonic cell lysates occurred whereas, and in stark contrast, substantially fewer and weaker bands were observed for biofilm cell lysates (Figure 2A). Not unexpectedly, considerable variability was observed between human serum samples with those from patient 2 and 3 having the most dramatic reduction in the ability to detect biofilm cell lysates. The opposite effect was observed with sera obtained from biofilm-immunized mice. Mouse antisera strongly recognized proteins in the biofilm cell lysates and was weakly reactive with cell lysates from planktonic pneumococci (Figure 2B). These findings demonstrate that the humoral immune response developed against one growth phenotype is indeed poorly reactive against the other due to altered protein production.

### Identification of proteins produced during biofilm growth that are recognized by convalescent sera

**Figure 3:**
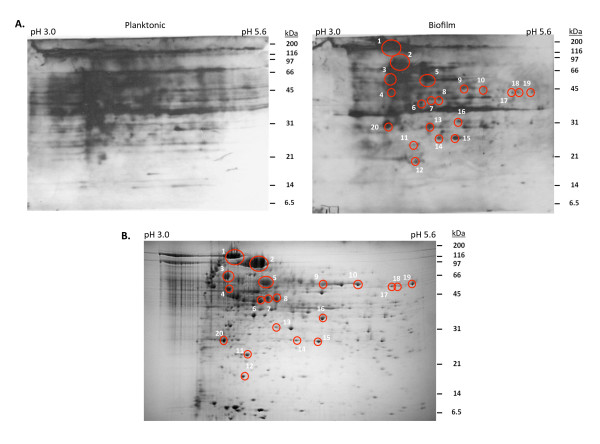
**Identification of immunogenic proteins enhanced during pneumococcal biofilm growth**. **A) **Immunoblots of planktonic and biofilm *S. pneumoniae *cell lysates separated by 2DGE and probed with pooled human convalescent sera. **B) **Coomassie blue stained 2DGE gel of biofilm proteins showing the 20 immunogenic protein spots (circled in red) selected for analysis by MALDI-TOF. The corresponding spots detected with convalescent sera are circled in the biofilm immunoblot in panel A.

As antigenic proteins produced during biofilm formation may represent novel targets for intervention, we identified pneumococcal proteins enhanced during biofilm growth that were also reactive with human convalescent sera. To do so, planktonic and biofilm whole cell lysates were separated by 2DGE and Western blotting was performed with pooled convalescent sera. Consistent with our previous immunoblots, 2DGE-transferred membranes with biofilm cell lysates were less immunoreactive than those loaded with planktonic cell lysates when probed with the convalescent human sera (Figure 3A).

By comparing the biofilm 2DGE immunoblots to their corresponding 2DGE Coomassie blue stained gels, we identified 20 protein spots enhanced during biofilm growth that were also immunoreactive (Figure 3B). These spots were excised and a total of 24 proteins were identified by MALDI-TOF mass spectrometry (Table 2). Twelve of these 24 proteins had been previously observed to be produced at lower levels during biofilm growth in the analysis of whole cell lysates (Table 1); a finding reflecting the fact that multiple proteins may be present within each 2D-gel spot. Of the remaining 12 proteins only PsrP had been detected as biofilm-growth enhanced during our previous MALDI-TOF analysis (Table 1). The remaining 11 proteins had varied roles in assorted housekeeping cellular processes.

**Table 2:** Biofilm proteins present in spots reactive with human convalescent sera identified by MALDI-TOF analyses

Gene Product	Annotation*
elongation factor G (*fusA*)	SP_0273*
alcohol dehydrogenase (*adhP*)	*SP_0285*
trigger factor (*tig*)	*SP_0400*
3-oxoacyl-(acyl carrier protein) synthase II	*SP_0422*
phosphoglycerate kinase (*pgk*)	*SP_0499*
molecular chaperone DnaK (*dnaK*)	SP_0517*
phenylalanyl-tRNA synthetase subunit beta (*pheT*)	SP_0581*
fructose-bisphosphate aldolase	SP_0605*
50S ribosomal protein L1	SP_0631*
pyruvate oxidase (*spxB*)	SP_0730*
branched-chain amino acid ABC transporter, amino acid binding protein (*livJ*)	*SP_0749*
30S ribosomal protein S1 (*rpsA*)	*SP_0862*
6-phosphofructokinase (*pfkA*)	SP_0896*
pyruvate kinase	*SP_0897*
hypothetical protein SP_1027	*SP_1027*
phosphopyruvate hydratase (*eno*)	SP_1128*
50S ribosomal protein L10 (*rplJ*)	SP_1355*
GMP synthase (*guaA*)	SP_1445*
NADH oxidase	*SP_1469*
F0F1 ATP synthase subunit alpha	SP_1510*
phosphoglyceromutase (*gpmA*)	SP_1655*
Pneumococcal Serine-rich repeat protein (*psrP*)	SP_1772*
acetate kinase	*SP_2044*
elongation factor Ts (*tsf)*	SP_2214*

* Identified in comparative analysis of biofilm versus planktonic lysates (Table 1).

### Immunization with biofilm-pneumococci does not protect against disease by other serotypes

**Figure 4:**
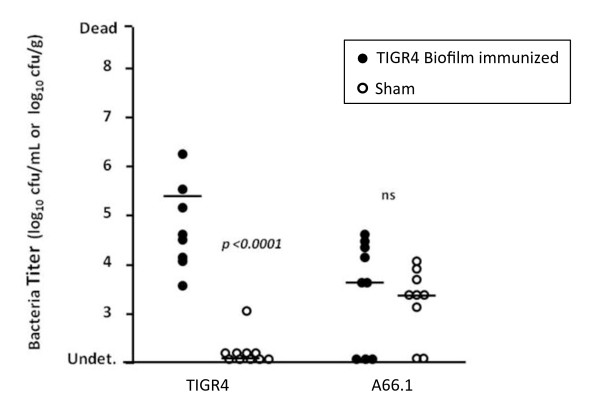
**Challenge of mice immunized with TIGR4 biofilm pneumococci**. Bacterial titers in the blood of mice challenged intranasally with 10^7 ^CFU of planktonic TIGR4 or A66.1 after 48 hours. Mice were immunized with ethanol-killed biofilm pneumococci in Freund's adjuvant (TIGR4 n = 8, A66.1 n = 9) or were sham-immunized and received Freund's adjuvant alone (TIGR4 n = 9, A66.1 n = 9). Each spot represents an individual mouse. Horizontal bars indicate the median value. Statistical analysis was performed using a two-tailed Student's *t*-test.

Finally, we tested whether immunization with ethanol-killed biofilm pneumococci conferred protection against challenge with the same strain or another belonging to a different serotype (Figure 4). Compared to sham-immunized control mice, animals immunized with TIGR4 biofilm cell lysates were protected against the development of bacteremia following challenge with TIGR4. In contrast, no protection was observed for mice challenged with A66.1, a serotype 3 isolate, despite prior immunization with TIGR4. Of note, A66.1 does not carry PsrP (data not shown). The protection observed against TIGR4 was most like due to the fact that the TIGR4 biofilm cell lysates, despite having a different protein profile, contained serotype 4 capsular polysaccharide, a protective antigen. Thus, immunization with biofilm-derived cell lysates was insufficient to confer protection against virulent pneumococci belonging to a different serotype.

## Discussion

Biofilms are recognized as the primary mode of growth of bacteria in nature. Notably more than half of all human bacterial infections are believed to involve biofilms [16,18]. Consistent with this notion, *S. pneumoniae *has been observed to form biofilms both *in vitro *and *in vivo *[9,12-14,24,30,33,34]; although during invasive disease, pneumococci in the bloodstream and sputum seem to be exclusively diplococci. While a large body of work has been published on the characteristics of pneumococcal biofilm formation *in vitro *as well as the genes involved in this process, little is known about the host immune response to pneumococcal biofilms and how this differs with respect to planktonic bacteria. This is a significant lapse as pneumococcal biofilms are now recognized to be present in the nasopharynx of colonized humans.

In the present study, we identified the differential protein profile of *S. pneumoniae *serotype 4, strain TIGR4 in a mature 3-day old biofilm versus during planktonic exponential growth. As expected, we observed considerable differences in the protein profiles of planktonic and biofilm TIGR4 with the vast majority of detected proteins being produced in diminished quantities. Notably, our proteomic findings are in disagreement with those of Allegrucci *et al*. which described a dramatic increase in the number of detectable proteins in 9 day-old biofilms including phosphoglyceromutase, phosphoglycerate kinase, 30S ribosomal protein S1, translation elongation factor Tu, 50S ribosomal protein L1, enolase, DnaK protein, and pyruvate oxidase, among many other proteins [24]. This discrepancy may be due to the different strains used, the different age of the biofilms examined, alternatively, due to our strict criteria for protein identification combined with the fact that that a large portion of mature biofilm is composed of dead and presumably degraded bacterial components. Importantly, our findings are in agreement with the generally accepted notion that the synthetic and metabolic activity of bacteria are reduced during biofilm growth [15,16], as well as with previous studies examining the transcriptional changes incurred during pneumococcal biofilm growth which showed down-regulation of the genes encoding many of these proteins [17,25,30,35].

Due to the altered protein profiles, unsurprisingly, but also previously undocumented, convalescent sera only robustly recognized planktonic cell lysates. Likewise, sera from biofilm-immunized mice weakly recognized cell lysates from planktonic pneumococci. Together, these results support the notion that invasive pneumococcal disease is predominantly caused by the planktonic phenotype. They also suggest that the antibody response and potentially the T-cell response generated against *S. pneumoniae *during nasopharyngeal colonization would be of limited utility against planktonic bacteria during invasive disease. This latter notion is supported by our finding that immunization with ethanol-killed TIGR4 biofilm pneumococci failed to protect against invasive disease caused by a serotype 3 isolate. In regards to the development of a protein vaccine using pneumococcal antigens, our findings strongly advocate that candidate proteins be explored for differences in production during biofilm and planktonic growth, which could affect an antigen's utility as a protective epitope.

The biofilm upregulated proteins that were reactive with convalescent sera included PsrP. Similar to our own findings, *Geifing et al*., found in an unbiased screen that recombinant PsrP also interacted with human convalescent sera [36], indicating that PsrP is also produced *in vivo *during invasive disease. The latter most likely reflects the dual role of PsrP as a bacterial and lung cell adhesin. Importantly, antibodies against PsrP are capable of neutralizing biofilm formation and lung cell attachment *in vitro*. Furthermore, immunization with recombinant PsrP BR has been shown to protect against invasive disease caused by TIGR4 [14,26,27,37]. Unfortunately, epidemiological studies indicated PsrP is present in only 50-60% of all invasive isolates [38]. Its absence in A66.1 thereby helps to explain the lack of protection that was observed in mice immunized with biofilm TIGR4. Along this line, it would be worthwhile to confirm that immunization of mice with biofilm TIGR4 protects against challenge with a non-serotype 4 PsrP-positive strain.

The remaining proteins with enhanced biofilm production that were also reactive with convalescent sera might also be protective antigens. In support of this notion, Brady *et al*. has shown that immunization of rabbits with biofilm *S. aureus *protected against osteomyelitis in a rabbit model of infection [39]. While the vast majority of the proteins that we identified are involved in metabolism, recent studies have shown that enzymes involved in glycolytic metabolism, including enolase and fructose bisphosphate aldolase, as well ribosomal proteins are localized to the cell surface of *S. pneumoniae, S. agalactiae and S. pyogenes *and are capable of playing a role in virulence [40-42]. Notably, the majority of proteins within the *S. aureus *biofilm fraction that was recognized by sera from rabbits with osteomyelitis were also predominately involved in metabolism [39]. Thus, further studies are warranted to determine whether antibodies against these *S. pneumoniae *metabolic proteins might confer protection against colonization and possibly invasive disease.

Importantly, incomplete strain coverage by PsrP and other pneumococcal proteins that have been suggested to be vaccine candidates, along with limited efficacy for those that are conserved in all strains such as pneumolysin and CbpA, indicate two or probably three proteins would be minimally required for complete coverage in any efficacious protein vaccine formulation against *S. pneumoniae *[43].

## Conclusions

In all, our findings add to the considerable body of evidence that indicates biofilm pneumococci have dramatically altered phenotypes versus planktonic bacteria. Our studies advance this concept and demonstrate that this altered protein profile results in a skewed antibody response during invasive disease, and that biofilm bacteria do not elicit a strong-cross-reactive humoral response against planktonic bacteria. This latter suggests that the adaptive immune response developed towards biofilm bacteria during colonization would have restricted utility during invasive disseminated disease. Our studies also identify PsrP as one possible antigen that may confer protection against both colonization and invasive disease. The other proteins identified as enhanced during biofilm formation and immunogenic during invasive disease may also represent novel targets for intervention.

## Methods

All animal experiments were reviewed and approved by the Institutional Animal Care and Use Committee at The University of Texas Health Science Center at San Antonio under protocol number 09022x-34.

### Strain and bacterial growth conditions

*Streptococcus pneumoniae *strain TIGR4 is a serotype 4 clinical isolate whose genome has been sequenced and annotated [44]. A66.1 is a serotype 3 isolate that has also been previously described [24]. For planktonic growth, Todd Hewitt Broth (THB) was inoculated with overnight plate cultures and grown to mid-logarithmic phase (OD_620 _= 0.5; ~1.0 × 10^8 ^CFU/ml) at 37°C in 5% CO_2_. Mature biofilms were grown under once-through flow conditions as previously described [14]. Briefly, planktonic seed cultures were used to inoculate 1 meter long silicone tubing (0.89 mm internal diameter, Cole Parmer Inc., Vernon Hills, IL). Bacteria in the line were allowed to attach for 2 hours, after which the flow rate of THB was adjusted to 0.035 ml/minute. Biofilm derived bacteria were harvested after 3 days by pinching the tube along its entire length, thereby removing the bacterial cells.

### One and two-dimensional gel electrophoresis and differential protein analysis

For one-dimensional (1DGE) comparative analysis of proteins, whole cell lysates (25 μg) from the biofilm and planktonic pneumococci were separated by 12% sodium dodecyl sulfate polyacrylamide gel electrophoresis (SDS-PAGE) and silver stained using standard methods. Two-dimensional electrophoresis (2DGE) was conducted according to the principles of O'Farrell [45], and done using the optimized conditions for *S. pneumoniae *as previously described by Allegrucci *et al*. [24]. Briefly, planktonic and biofilm pneumococci were collected, washed, and suspended in TE buffer (10 mM Tris-HCl, 1 mM EDTA, pH 8.0) supplemented with 300 μg/ml phenylmethyslfonylfluoride (Sigma, St. Louis, MO). Bacteria were disrupted by sonication on ice using 6, 10-second bursts. Samples were prepared for isoelectric focusing (IEF) using a ReadyPrep 2-D cleanup kit (Bio-Rad, Hercules, CA) after which the protein pellet was dissolved in DeStreak rehydration solution (GE Healthcare, Piscataway, NJ). Protein levels were quantified using a Non-Interfering protein assay (G-Biosciences, Maryland Heights, MO). For each sample, 300 μg of protein were applied to 11-cm Immobiline DryStrips (pH 3-5.6 Non-linear, GE Healthcare) and rehydrated for 17 hours at 4° C with DeStreak rehydration solution containing 0.5% IPG buffer (pH 3-5.6 NL, GE Healthcare). The rehydrated IPG strips were focused at 20°C for a total of 17 kVh using an Ettan IPGphorII IEF system (GE Healthcare). Prior to the separation by SDS-PAGE, IPG strips were equilibrated using a reducing buffer (75 mM Tris-HCI, pH 8.8), 6 M urea, 29.3% glycerol, 2% SDS, 1.0% dithiothreitol, and 0.002% bromophenol blue) for 15 minutes at room temperature, followed by alkylation with 2.5% (wt/vol) iodoacetamide for an additional 15 minutes. Proteins were separated on pre-cast 8-16% gradient Criterion polyacrylamide gels at 200 V (Bio-Rad, Hercules, CA). Protein spots were visualized by Coomassie blue staining, and gel images were recorded using a ChemiDoc XRS system (Bio-Rad).

### Antiserum against *S. pneumonia*

Convalescent serum from 3 individuals recently recovered from confirmed pneumococcal pneumonia was a kind gift from Dr. Daniel Musher (Houston, TX). Antibodies against biofilm pneumococci were generated in 6 week old female Balb/c mice by immunization with 20 μg of ethanol-killed biofilm pneumococci emulsified with Freund's Complete Adjuvant (Sigma). After 21 and 42 days, mice were boosted with the same bacterial sample emulsified with Freund's Incomplete Adjuvant (Sigma). Sera from vaccinated mice were collected at day 50 by retro-orbital bleeding.

### Western blotting

1D and 2D gels were electrophoretically transferred to nitrocellulose membranes, blocked in PBS containing 4% bovine serum albumin (BSA) and 0.1% Tween-20 (T-PBS) for 1 hour and incubated overnight at 4 °C with T-PBS containing convalescent sera (1:10,000) from each of the individual patients or from immunized mice. Following overnight incubation, membranes were washed 3 times with T-PBS for 5 minutes and a secondary HRP-conjugated Goat anti-human IgG antibody (Sigma) (1:5,000) or Goat anti-mouse IgG antibody (Jackson Immunoresearch Laboratories, Westgrove, PA) was used for detection of the immunogenic proteins recognized by human convalescent sera or sera from immunized mice by chemiluminesence respectively.

### Protein identification by mass spectrometry

Proteins of interest were excised from SDS-PAGE gels and destained twice in 50% acetonitrile (ACN)/40 mM ammonium bicarbonate (pH 7.4), prior to digestion. Gel plugs were then dehydrated in 100% ACN and rehydrated with 5-10 μl of 10 ng/μl trypsin (Promega, Madison WI) in 40 mM ammonium bicarbonate/20% ACN and incubated overnight at 30° C. Peptides were extracted in 4 volumes of 0.1% trifluoroacetic acid (TFA) in 50% ACN for 1 to 2 hours at room temperature, decanted from the gel slice, dried down in an autosampler tube in a speed vacuum without heat, and suspended in 0.1% TFA. Peptides were analyzed by capillary-HPLC-electrospray tandem mass spectrometry (HPLC-ESI-MS/MS) on a Thermo Fisher LTQ ion trap mass spectrometer coupled to an Eksigent NanoLC micro HPLC by means of a PicoView (New Objective, Woburn, MA) nanospray interface. Capillary on-line HPLC separation of tryptic peptides was conducted using the following conditions: column, New Objective PicoFrit, 75 μm id, packed to 11 cm with C18 adsorbent (Vydac 218MSB5); mobile phase A, 0.5% acetic acid/0.005% TFA in water; mobile phase B, 90% ACN/0.5% acetic acid/0.005% TFA in water; gradient, 2% B to 42% B in 30 min; flow rate, 0.4 μl/min. A data-dependent acquisition protocol was employed consisting of one survey scan followed by 7 collision-induced dissociation spectra. The un-interpreted CID spectra were searched against the NCBI NR database using Mascot (Matrix Science; 10 processor in-house license). Methionine oxidation was the only variable modification considered. Maximum missed cleavages for trypsin was set at 1, peptide charge at 2+ and 3+, peptide tolerance at +/- 1.5 Da, and MS/MS tolerance at +/- 0.8 Da. Mascot data was then run in Scaffold 3.1 http://www.proteomesoftware.com and cross-correlation of the Mascot results was carried out by X! tandem against the NCBI NR subset database. Proteins with an expectation score of 10^-3 ^or lower were considered positive identities. Proteins were identified with 3-15 matched peptides and a minimum of 95% sequence coverage.

### Mouse challenge experiments

At day 56, TIGR4 biofilm- and sham-immunized mice (i.e. receiving only Freund's adjuvant), were challenged intranasally with 10^7 ^CFU of planktonic TIGR4 or A66.1 in 25 μl PBS [37]. On day 2 post-infection, blood was collected from the tail vein of each mouse and bacterial titers determined by serial dilution, plating, and extrapolation from colony counts following overnight incubation. Statistical analysis was performed using a two-tailed Student's *t*-test.

## Authors' contributions

CJS and BJH carried out the isolation of protein lysates, 1DGE and 2DGE separations, and immunoblots. AL critically reviewed the MALDI-TOF data. PS developed antiserum against biofilm pneumococci. GTC and CJO participated in the design and coordination of the studies. All authors read and approved the final manuscript.

## Author's Information

None
